# Diagnostic pathways and direct medical costs incurred by new adult pulmonary tuberculosis patients prior to anti-tuberculosis treatment – Tamil Nadu, India

**DOI:** 10.1371/journal.pone.0191591

**Published:** 2018-02-07

**Authors:** Karun Sandeep Veesa, Kamalabhai Russell John, Patrick K. Moonan, Saravanakumar Puthupalayam Kaliappan, Krishna Manjunath, Karuna D. Sagili, Chinnappareddy Ravichandra, Pradeep Aravindan Menon, Chandrakumar Dolla, Nancy Luke, Kaivan Munshi, Kuryan George, Shantidani Minz

**Affiliations:** 1 Department of Community Health, Christian Medical College, Vellore, Tamil Nadu, India; 2 Department of Community Medicine, Apollo Institute of Medical Sciences, Chittoor, Andhra Pradesh, India; 3 Division of Global HIV and Tuberculosis, United States Centers for Disease Control and Prevention, Atlanta, Georgia, United States of America; 4 International Union against Tuberculosis and Lung Disease, The Union South-East Asia Office, New Delhi, India; 5 National Tuberculosis Institute, Bangalore, Karnataka, India; 6 National Institute for Research in Tuberculosis, Chennai, Tamil Nadu, India; 7 Department of Sociology and Criminology, Population Research Institute, The Pennsylvania State University, Pennsylvania, United States of America; 8 Department of Economics, University of Cambridge, Cambridge, United Kingdom; Johns Hopkins University Bloomberg School of Public Health, UNITED STATES

## Abstract

**Background:**

Tuberculosis (TB) patients face substantial delays prior to treatment initiation, and out of pocket (OOP) expenditures often surpass the economic productivity of the household. We evaluated the pre-diagnostic cost and health seeking behaviour of new adult pulmonary TB patients registered at Primary Health Centres (PHCs) in Vellore district, Tamil Nadu, India.

**Methods:**

This descriptive study, part of a randomised controlled trial conducted in three rural Tuberculosis Units from Dec 2012 to Dec 2015, collected data on number of health facilities, dates of visits prior to the initiation of anti-tuberculosis treatment, and direct OOP medical costs associated with TB diagnosis. Logistic regression analysis examined the factors associated with delays in treatment initiation and OOP expenditures.

**Results:**

Of 880 TB patients interviewed, 34.7% presented to public health facilities and 65% patients sought private health facilities as their first point of care. The average monthly individual income was $77.79 (SD 57.14). About 69% incurred some pre-treatment costs at an average of $39.74. Overall, patients experienced a median of 6 days (3–11 IQR) of time to treatment initiation and 21 days (10–30 IQR) of health systems delay. Age ≤ 40 years (aOR: 1.73; CI: 1.22–2.44), diabetes (aOR: 1.63; CI: 1.08–2.44) and first visit to a private health facility (aOR: 17.2; CI: 11.1–26.4) were associated with higher direct OOP medical costs, while age ≤ 40 years (aOR: 0.64; CI: 0.48–0.85) and first visit to private health facility (aOR: 1.79, CI: 1.34–2.39) were associated with health systems delay.

**Conclusion:**

The majority of rural TB patients registering at PHCs visited private health facilities first and incurred substantial direct OOP medical costs and delays prior to diagnosis and anti-tuberculosis treatment initiation. This study highlights the need for PHCs to be made as the preferred choice for first point of contact, to combat TB more efficiently.

## Introduction

Tuberculosis (TB), an airborne infectious disease, is one of the leading causes of mortality in India [1]. India is one of the 30 high burden countries for TB, TB-HIV and MDR-TB. More than one quarter of TB cases and TB-related deaths worldwide occur in India each year, the highest of any country [2]. The private sector is an important source of treatment of tuberculosis, where 5.3 million cases received treatment in 2014 alone [3]. Lack of protocol based diagnostic practices in the private sector delays accurate diagnosis, and the treatment offered is maybe substandard [4,5]. Patients are not aware of the national program [6] and meet several health care providers before receiving the correct diagnosis and appropriate treatment regimen [7,8]. It remains unclear to what extent these health seeking behaviours and substandard clinical practices result in more advanced disease progression, increased transmission, and financial burden to the patient.

TB primarily affects the poor. Those who develop TB often experience severe economic hardship. A study from rural India found that TB patients face substantial catastrophic costs associated with their illness; on average, the overall costs exceeded 193% of the estimated monthly income of a daily wage labour [9]. There is substantial evidence showing financial burden of TB in middle and low-income countries, where the average total costs incurred for TB treatment are equivalent to 58% of annual individual income and 39% of household income [10]. The out of pocket expenditure for medical care far surpasses the economic productivity of household. The World Health Organization (WHO) has set an ambitious target to eliminate the number of TB-affected families facing catastrophic costs associated with tuberculosis (e.g., exceeding 20% of the household’s annual income) by 2035 [11].

The purpose of this study is to evaluate the pre-diagnostic cost and to explore the patient health care-seeking pathways for diagnosis and treatment initiation. We describe the pathways (i.e., number and type of health facilities visited and time taken to start with treatment initiation) and direct OOP medical costs incurred prior to the initiation of anti-tuberculosis treatment of new adult pulmonary TB (PTB) patients under Revised National Tuberculosis Control Program (RNTCP) in India.

## Methods

### Study design and setting

The descriptive study was part of a larger randomized controlled trial (RCT) designed to test the efficacy of TB case management by different types of community based DOT providers from a cohort of TB patients recruited between December 2012 and December 2015. The study was conducted in rural areas of Vellore district, Tamil Nadu, a southern state of India. Vellore is one of the larger districts in Tamil Nadu, where 57% of the population lives in rural areas [12]. The public health care facilities in the district include 101 primary health centres (PHCs), 13 secondary referral hospitals (Sub district hospitals), and one tertiary referral hospital (Government Vellore Medical College). Tuberculosis services are provided through 7 Tuberculosis Units (TUs). Apart from these, there are private health care facilities, one tertiary care centre (Christian Medical College and Hospital), private hospitals, private practitioners, and those practising Ayurveda, Yoga, Unani, Siddha and Homoeopathy (AYUSH) systems of medicine.

Out of 7 TUs in Vellore district, the parent study purposively selected three peripheral TUs (Natrampalli, Pudhupadi, and Punnai) because these are geographically distant from the tertiary health care facilities and that would best reflect TB case management among the rural population.

### Study participants

Of the 1833 patients registered for DOTS in 48 PHCs during the study time period, the parent study enrolled 1080 adult PTB cases (923 new and 157 previously treated cases) after meeting eligibility criteria and informed consent. The study excluded patients with severe illnesses, such as weight less than 30 kg, acute or chronic liver or kidney disease, psychiatric illness, history of default on previous DOTS based on medical record review, and non-ambulatory patients, defined as persons not being able to go to the PHC/DOT provider’s house by usual transport. Of the 923 new cases, we were able to identify 880 patients’ (95%) treatment pathways, direct OOP medical costs, and time delays information. These 880 patients were included in the secondary analysis.

### Ethics statement

The parent study, RCT has been approved by the Institutional Review Boards of Brown University, USA; Christian Medical College, Vellore, India; Pennsylvania State University, USA; University of Cambridge, UK; and the National Institute for Research in Tuberculosis, Chennai, India. The study was registered in Clinical Trials Registry of India (CTRI/2016/08/007203). The present secondary analysis of data was approved by the Union Ethics Advisory Group, Paris, France. Participation of the US Centers for Disease Control and Prevention (CDC) in this project did not meet the definition of engagement in human subjects’ research and hence a separate institutional review board approval was not required.

### Operational terms

#### Pathways

Patient visits to various health facilities in a care pathway before anti-tuberculosis treatment initiation at DOTS centre (facility in the health system for the diagnosis and treatment of TB under national TB control program, RNTCP) were classified in to two broad categories: 1) Public and 2) Private (PVT). Public health care facilities were further categorized in to: A) Primary Health Centres (PHCs), B) Public Referral Hospitals (PRHs)—including secondary and tertiary referral centres, and C) TB Sanatorium (ST), a nodal centre for TB management, situated in Tambaram, Chennai, about 120 km away from the study area. Private health care facilities include secondary and tertiary referral centre participating in RNTCP, non-participating allopathic clinics and practitioners (registered and unregistered) and AYUSH practitioners.

#### Direct medical costs

Direct medical costs included out of pocket payment (OOP) incurred by the patient for the diagnosis of illness in health facilities before treatment initiation. This includes a composite cost of consultation fees, investigations (such as Haemoglobin, Total, and differential counts, Blood sugar, Liver function test, Serum creatinine, Sputum for acid fast bacilli, Chest X-ray, and additional tests such as CT-Scan) and cost of all medicines prescribed at the time of health facility visits.

#### Time delays in a care pathway

**Fig 1:**
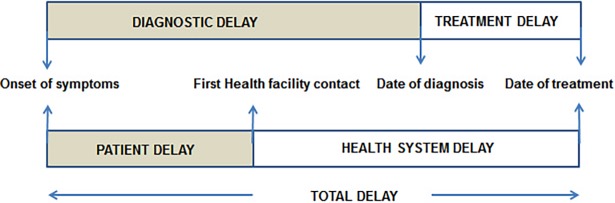
Framework showing various time delays from onset of symptoms to treatment initiation. Study presented only health system and treatment delays (Unshaded areas).

The framework of time delays was adopted from a WHO study “Diagnostic and treatment delay in tuberculosis” [13] (Fig 1). We studied two time delays in a care pathway: 1) *Health systems delay*–defined as the duration from first health care facility visit to date of anti-tuberculosis treatment initiation, and 2) *Treatment delay*–defined as the duration of greater than 7 days between the date of TB diagnosis and date of anti-tuberculosis treatment initiation under DOTS in respective PHCs [14].

Current smoker is someone who has smoked more than 100 cigarettes or hand rolled beedis in their lifetime and has smoked in last 28 days. Current alcohol user is someone who had a past history of alcohol consumption and has consumed in last 28 days.

### Data collection

Trained field investigators collected the data using structured questionnaire in a direct interview. Interviews were conducted either by making home visits or at the health centres based on study participants’ preference. Participants were then asked for information related to past experiences at different health facilities, incurred direct OOP medical costs for each facility, and the time taken to start anti-tuberculosis treatment. Further information related to the place of diagnosis and date of treatment initiation was recorded from the TB programme registers at PHCs.

### Data analysis

Data collected was double entered and analysed using STATA.13 (StataCorp College Station, TX). Key clinical and demographic patient characteristics were summarized using means (standard deviation, SD) and medians (inter quartile range, IQR). Direct OOP medical costs and health systems delay were dichotomised by median cut-off values. We used univariable analysis to describe the distribution of select risk factors (e.g., age, sex, education, employment status, health behaviours, body mass index (BMI), and HIV infection and other comorbid conditions). We used multiple logistic regression to assess factors independently associated with the primary outcomes–median of direct OOP medical costs and health systems delay–based on adjusted odds ratios (aOR) and corresponding 95% confidence intervals (95% CI). All potential models were reduced using backward elimination, maintaining variables with *P* values <0.05.

### Economic analysis

Cost data was collected using local currency units (Indian Rupees, INR) directly reported from participants. All local currency amounts were converted into 2017 US $ [$1.00 = 64.66 INR].

## Results

### Patient characteristics

Of 880 patients (685 initial sputum smear-positive and 195 sputum smear-negative), 688 (78%) were males and 192 (22%) were females, with an average age of 46.6 (SD 14.16) years. The majority of patients (78%) did not attain a secondary level of education. Among the 696 (79%) employed population, a large proportion were daily wage earners either in agriculture or industrial sectors. The average monthly individual income was $77.79 (SD 57.14). Of 688 males, 58 (6.5%) were smokers, 137 (15.5%) were alcohol consumers, 204 (23%) undertook both behaviours and 481(55%) do not smoke or consume alcohol. Of 192 females, there were two smokers, four alcohol consumers and one undertook both behaviours. The majority of patients (69%) were underweight (BMI <18.5) at the time of treatment initiation.

### Health care seeking patterns in the patient care pathway

**Fig 2:**
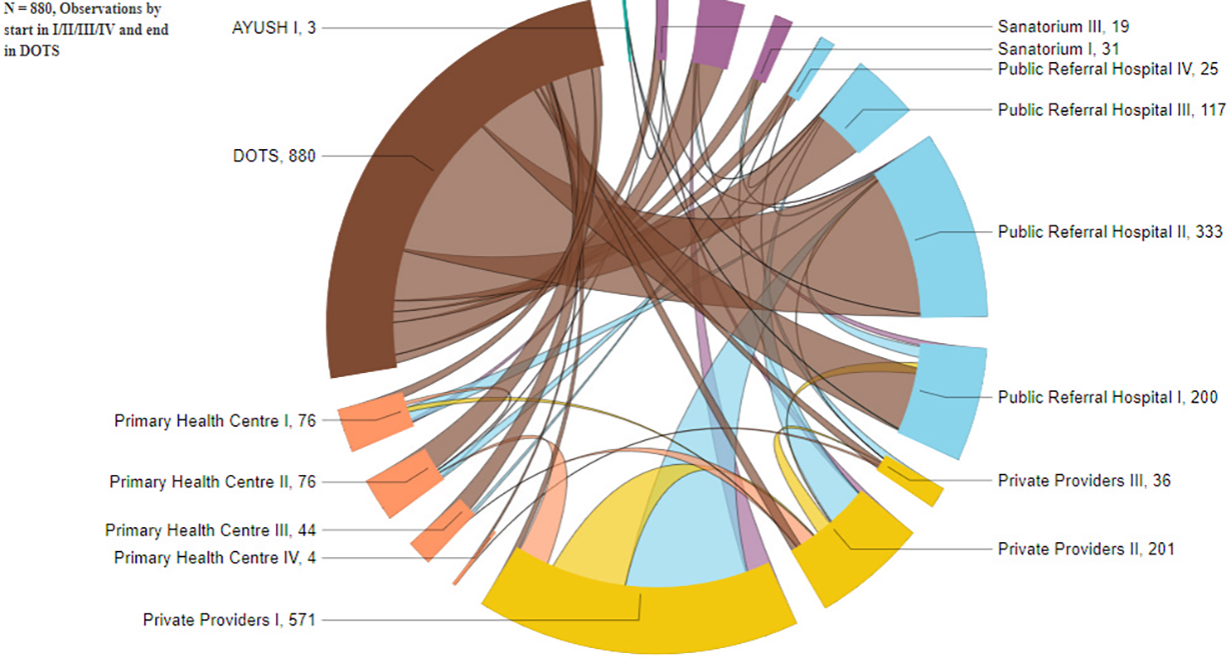
Chord diagram showing patients’ pathways to different health facilities prior to DOTS initiation in primary health centres under RNTCP, India. Chord diagram shows the inter-relationships between patient’s pathways to different health facilities in order of I/ II/ III/ IV towards DOTS, the volume at each facility is presented in numbers and the matrix with different colours indicates the pathway relationship. Interactive link https://app.powerbi.com/view?r=eyJrIjoiM2FjNzJiMmUtNDVjNy00YTNkLTgwZjMtNGQzOTI0NzYwOTk0IiwidCI6IjdlNzgyYTc2LWUzZTQtNDQ1Ny04YzQzLWQ1NDVjNmRkYjUwZSJ9.

The study recorded a maximum of four health care facility visits by patients prior to anti-tuberculosis treatment initiation. The 880 patients’ pathways to different health care facilities (Fig 2) were divided into 20 major care pathways (Table 1). We found that 306 (35%), 571 (65%) and 3 (0.3%) patients first sought treatment at public, private, and AYUSH health care facilities, respectively. Of the 306 patients who sought treatment first at public health care facilities, 207 (68%) started on treatment whereas of the 571 patients who went to private health care facilities first, only 63 (11%) were started on treatment. The remaining made visits to a myriad of other health facilities before treatment initiation. Furthermore, these patients who visit private health facilities first were more likely to have multiple visits to other providers. The majority of patients (91%) were diagnosed at a public health facility finally. Overall, the largest group (55%) was diagnosed at secondary referral centres (Sub district hospitals) and few at private health facilities (9%) or PHCs (10%) (Table 2).

**Table 1:** Pathways, pre-diagnosis direct OOP medical costs, and health systems delay among new adult pulmonary TB patients registered for DOTS in rural PHCs, Vellore district, India.

Pathways	n	%	Direct OOP medical costs in US $ Median (IQR)	Health systems delay (in days) Median (IQR)
**First health visit in PHC to DOTS**				
PHC	21	2.4	0.00	10 (7–21)
PHC → PRH	34	3.9	0.00	14 (8–30)
PHC → ST	10	1.1	0.00	28 (17–52)
PHC → PVT	4	0.5	7.70 (4.20–39)	16 (15–20)
PHC → PVT → PRH/ST	7	0.8	31 (17–93)	20 (17–31)
Total first visit in PHC	76	8.7	0.00	17 (10–30)
**First health visit in PRH to DOTS**				
PRH	162	18.4	0.00	14 (7–30)
PRH → ST	12	1.4	0 (0–0.50)	30 (22–67)
PRH → PVT	16	1.8	23 (12–71)	25 (12–30)
PRH → PVT → PRH/ST	9	1.0	8.30 (7.70–12.40)	18 (14–30)
Total first visit in PRH	199	22.6	0.00	14 (7–30)
**First health visit in ST to DOTS**				
ST	24	2.7	0.00	30 (13–30)
ST → PRH	6	0.7	0.00	33 (14–60)
ST → PVT → PRH	1	0.1	15.50	18
Total first visit in ST	31	3.5	0.00	30 (14–30)
**First health visit in PVT to DOTS**				
PVT	63	7.2	12.40 (5.80–31)	14 (7–30)
PVT → PRH	268	30.5	9.30 (4.40–18.60)	21 (14–30)
PVT → ST	70	8.0	11.60 (6.30–26)	30 (14–36)
PVT → PVT	60	6.8	43.80 (15.50–77.50)	30 (14–30)
PVT → PHC → PRH	17	1.9	7.70 (6.20–46.50)	20 (10–30)
PVT → PVT → PRH	76	8.6	31 (15.50–59.60)	30 (15–45)
PVT → PVT → PVT → PRH	17	1.9	77.50 (44–248)	30 (14–60)
Total first visit in PVT	571	65	13 (6.20–38.70)	25 (14–30)
**First health visit in AYUSH to DOTS**				
AYUSH	3	0.3	31 (15.50–38.70)	30 (22–45)
**First health visit in Public / Private health system / AYUSH to DOTS**				
First visit in Public health sector (PHC+PRH+ST)	306	34.7	0.00	15 (7–30)
First visit in Private health sector (PVT)	571	65	13 (6.20–38.70)	25 (14–30)
First visit in AYUSH	3	0.3	31 (15.50–38.70)	30 (22–45)
Overall	880	100	7.75 (0–23.20)	21 (10–30)

PHC–Private Health Centres, PRH–Public Referral Hospitals (secondary and tertiary referral centres), ST–Sanatorium, PVT–Private health facilities, AYUSH—Ayurveda, Yoga, Unani, Siddha and Homoeopathy systems of medicine

**Table 2:** Place of TB diagnosis and time taken for treatment initiation after diagnosis (treatment delay, if > 7 days).

Place of TB diagnosis	N (%)	Time taken for treatment initiation Median (IQR) in days	Treatment delay Median (IQR) in days	Treatment delay (>7 days) n (%)
Primary health centres (PHC)	88 (10)	5 (2–9)	14 (9–20)	33 (38)
Public secondary referral centres^a^	486 (55)	5 (2–8)	12 (9–18)	125 (26)
Public tertiary referral centres^b^	78 (9)	6 (4–10)	11 (9–16)	28 (36)
Private hospitals/clinics^c^	30 (3)	4 (2–14)	17 (14–32)	11 (37)
Private tertiary referral centres^b^	49 (6)	8 (4–12)	12 (9–18)	25 (51)
TB Sanatorium^d^	149 (17)	12 (7–19)	15 (11–22)	109 (73)
Total	880	6 (3–11)	13 (10–20)	331 (38)

^a^Public secondary referral centres include all the sub district hospitals in the study area.

^b^Tertiary referral centres include the medical colleges where patients were diagnosed and referred to DOTS centre

^c^Private hospitals/clinics include secondary hospitals and private practitioners in the study area

^d^TB Sanatorium is a higher centre for the TB management, the observed delay in treatment initiation was due to delay in transit from TB sanatorium to the respective PHCs for registration under DOTS.

### Direct out of pocket medical expenditure in stages of patient care pathways

The median/IQR of out of pocket payments per patient was $7.75 ($0.00 –$23.20), with a mean of $27.30. About one third (31%) of patients did not incur any pre-treatment costs. Of the 69% who incurred pre-treatment costs, the average amount spent was $39.74. The average cost for patients who visited private health care facilities first was $38.78. When we compared the costs incurred by patients who visited private and public health sectors, patients who visited private health facilities first spent more: Median(IQR) $13.00 ($6.20 –$38.70) vs. $0.00 ($0.00 - $0.00) in public health facilities (Table 1).

### Heath systems delay and treatment delay in a patient care pathway

The median number of days taken for initiation of anti-tuberculosis treatment from the day of first visit to a health facility (health systems delay) was 21 days (10–30 IQR) (Table 1). The minimum and maximum days taken for initiation of anti-TB treatment was 1 and 365 days. The median and interquartile range values for health systems delay in various facilities were 17 days (10–30 IQR) at PHCs, 14 days (7–30 IQR) at PRHs, 30 days (14–30 IQR) at the TB Sanatorium and 25 days (14–30 IQR) days at PVT health facilities. Very few patients, 3 (0.3%), visited AYUSH practitioners, and the median days to initiation of treatment was 30 days (22–45 IQR).

We observed a time lag between the point of diagnosis of illness and treatment initiation was a median of 6 days (3–11 IQR). After diagnosis of illness, 38 (4%) patients were started on treatment on the day of diagnosis, 511 (58%) patients were started treatment between one to seven days and 331 (38%) patients started treatment after seven days (our definition of treatment delay). The majority of patients 486 (55%) were diagnosed at secondary referral hospitals and had a median of 5 days (2–8 IQR) duration between point of diagnosis of illness and treatment initiation. Of the 149 (17%) patients diagnosed at TB Sanatorium, 109 (73%) experienced a median treatment delay of 15 days (11–22 IQR) (Table 2).

Direct OOP medical costs that exceeded the median were associated with age (≤ 40 years), secondary level of education, current employment, non-smoking status at time of treatment initiation, diabetes, low BMI, and first visit to a private health facility in a care pathway. After adjusting for the above in logistic regression analysis, age (≤ 40 years), diabetes, and first visit to private health facility were significantly associated with higher pre-treatment OOP cost (Table 3).

**Table 3:** Factors associated with high direct out of pocket medical costs (above median 7.75 US $) in a patient care pathway by univariable and multivariable analysis.

Characteristics	n	High direct OOP medical costs, n (%)	Unadjusted odds ratio (95% CI)	Adjusted odds ratio (95% CI)
***Sex***				
Male (R)	688	307 (77)		
Female	192	94 (23)	1.19 (0.86–1.63)	——
***Age*, *years***				
>40 (R)	566	239 (60)		
≤ 40	314	162 (40)	1.45 (1.10–1.92)	1.73 (1.22–2.44)
***Education***				
Less than secondary (R)	685	300 (75)		
Secondary and above	189	99 (25)	1.41 (1.02–1.95)	1.19 (0.79–1.79)
***Employment status***				
Non–working (R)	184	72 (18)		
Working	696	329 (82)	1.39 (1.00–1.94)	1.39 (0.94–2.06)
***Current smoker***				
Yes	265	107 (27)	0.73 (0.55–0.99)	0.83 (0.57–1.20)
No (R)	615	294 (73)		
***Current alcohol user***				
Yes	346	156 (39)	0.96 (0.73–1.27)	——
No (R)	534	245 (61)		
***HIV–TB***				
Yes	32	14 (6)	1.36 (0.67–2.77)	——
No (R)	848	239 (94)		
***Diabetes***				
Yes	176	104 (26)	1.97 (1.41–2.76)	1.63 (1.08–2.44)
No (R)	704	297 (74)		
***BMI***				
Underweight (R)	597	251 (65)		
Normal & above	266	137 (35)	1.46 (1.09–1.95)	1.30 (0.90–1.86)
***Form of TB***				
Pulmonary smear positive (R)	685	305 (78)		
Pulmonary smear negative	179	88 (22)	1.20 (0.86–1.67)	——
***First health care facility visit***				
Public (R)	307	29 (7)		
Private	573	372 (93)	17.7 (11.6–26.9)	17.2 (11.1–26.4)

Longer health system delays (above median 21 days) were associated with age (> 40 years), diabetes, and those who first visited private health facility in a care pathway. After adjusting for the above in logistic regression analysis, age (> 40 years) and first visit to private health facility were significantly associated with health system delay (Table 4). There were no associations between gender, occupation, initial smear status, current smoker, and alcohol consumption with health system delays and incurred direct OOP medical costs in patient care pathways.

**Table 4:** Factors associated with health systems delay (above median 21 days) in a patient care pathway by univariable and multivariable analysis.

Characteristics	n	Health systems delay, n (%)	Unadjusted odds ratio (95% CI)	Adjusted odds ratio (95% CI)
***Sex***				
Male (R)	688	305 (77)		
Female	192	93 (23)	1.15 (0.83–1.58)	——
***Age*, *years***				
>40 (R)	566	276 (69)		
≤ 40	314	122 (31)	0.65 (0.49–0.86)	0.64 (0.48–0.85)
***Education***				
Less than secondary (R)	685	319 (80)		
Secondary and above	189	76 (20)	0.76 (0.54–1.05)	——-
***Employment status***				
Non–working (R)	184	72 (18)		
Working	696	329 (82)	1.33 (0.95–1.86)	——-
***Current smoker***				
Yes	265	124 (31)	1.10 (0.82–1.47)	——
No (R)	615	274 (69)		
***Current alcohol user***				
Yes	346	166 (42)	1.22 (0.92–1.60)	——
No (R)	534	232 (58)		
***HIV–TB***				
Yes	32	11 (4)	0.96 (0.47–1.98)	——
No (R)	848	270 (96)		
***Diabetes***				
Yes	176	94 (24)	1.50 (1.07–2.09)	1.26 (0.89–1.78)
No (R)	704	304 (76)		
***BMI***				
Underweight (R)	597	262 (68)		
Normal & above	266	126 (32)	1.15 (0.86–1.54)	——
***Form of TB***				
Pulmonary smear positive (R)	685	303 (78)		
Pulmonary smear negative	179	87 (22)	1.21 (0.87–1.69)	——
***First health care facility visit***				
Public (R)	307	110 (28)		
Private	573	288 (72)	1.77 (1.33–2.36)	1.79 (1.34–2.39)

## Discussion

The Government of India’s National Strategic Plan (NSP 2017–2025) aims to achieve “zero” catastrophic cost for TB patients by 2020 and the elimination of TB by 2025 through an integrated four-strategies approach: “Detect–Treat–Prevent–Build” (DTPB) [15]. Our study highlighted the patients’ treatment pathways and burden of direct OOP medical costs prior to DOTS initiation under the national TB control program (RNTCP) in India. The study exclusively examined a rural population where health care seeking is onerous compared to urban areas. We found that two thirds of patients registered under DOTS in PHCs had visited private health care facilities first and their OOP (direct medical) payments were 17 times higher and their health system delays were 1.8 times more likely to be long (above the median 21 days) than the patients who first approached the public health system.

A study by Rajeswari *et al*, conducted before the large-scale implementation of DOTS in Tamil Nadu, reported that 54%, 27% and 19% of TB patients first consulted private practitioners, public health systems and others, respectively, for their TB diagnosis and treatment [7]. A contemporary qualitative study by Sudha *et al* conducted in southern India found that private practitioners were the first and preferred point of contact for 57% of urban and 47% of the rural populations, and the major reasons quoted were the good quality of care and proximity of a health facility to the residence [16]. After implementation of DOTS, studies from southern India by Selvam *et al* and Charles *et al* reported 47% and 61% of TB patients in rural areas sought first treatment at public health facilities, respectively [8] [17]. In contrast, after a decade of DOTS in Tamil Nadu, our study documented that only 35% of rural TB patients first sought treatment in the public health system. This observed difference could be due to purposive selection of study TUs by the parent study, varying programmatic conditions, or sample selection (our sample consisted of TB patients eventually treated in the public health system). However, studies done in other regions of India have reported lower proportion of TB patients visiting public health system first (Karnataka (16%) and rural Maharashtra (19%)) [18,19].

The private sector in India treats a large number of TB patients; as many as 5 million patients were treated in 2014 alone [3]. This supports the view that, despite substantial planning, investment, and implementation of the TB program through the public health system, the private sector continues to attract a large proportion of TB patients [20]. Moreover, the quality of TB care offered by private practitioners is questionable. A recent systematic review on quality of TB care in India finds suboptimal care [5].

Regarding the pre-treatment economic burden to the patient, several studies have reported that the majority of costs were incurred during pre-diagnosis and the intensive phase of treatment—the most critical stages of a TB episode [21,22]. We observed that 69% of patients enrolled in the study who underwent treatment under public health system, incurred some direct OOP medical costs during pre-treatment. On an average, these patients spent 2570 INR ($39.74), half the average monthly individual income. Substantial OOP direct medical costs were incurred by patients who first approached private health facilities for the diagnosis of illness compared to those who presented directly to the public health system, where median costs were zero. Despite the availability of free TB diagnostic services under the public health system, most patients chose a private provider first resulting in substantial spending for diagnosis and treatment [23], which imposes an economic burden to the patient’s household [9].

Time delays occur at every point along the complex health care pathways followed by TB patients for diagnosis and treatment [24]. Similar to previous studies [7,25,26], we found that patients who initiated treatment in the private sector had a higher number of health facility visits compared to those who first visited public health facilities. Patients who visited private health care facilities initially also had longer health system delays: a median of 25 days (14–30 IQR) compared to 15 days (7–30 IQR) of health system delay for those who began in public health facilities. A greater proportion of delays occurred among the patients who visited the TB sanatorium—a median of 30 days (14–30 IQR) of health system delay and 15 days (11–22 IQR) of treatment delay. The sanatorium is a higher centre for the TB management and the delays should be negligible. The lengthy treatment delay could be due to the transit time needed to reach the respective PHCs for DOTS registration. Though the patients were immediately started on anti-tuberculosis treatment at the sanatorium, they are directed to register at their respective PHCs to continue the treatment. Our study considered the date of registration in DOTS centre at respective PHCs as the date of initiation of treatment, since the drug box was issued in the name of patient on this date.

We observed a median health system delay of 21 days (10–30 IQR) and the maximum was 365 days. The magnitude of health systems delay observed in other studies in different settings in India ranged from a median of 13 to 54 days [18,19,27,28] and a median of 5 to 22 days in other countries [29–31]. Delays in treatment initiation further increase the probability of TB transmission in the community and amongst health professionals. The observed differences across studies could be due to heterogeneity in definitions of delays (several studies define first health care visit to date of diagnosis as health systems delay, whereas other studies consider the time to the treatment initiation), complexity in study settings [25], or differences in treatment procedures at health facilities.

In the study we observed only few private hospitals/clinics were linked to DOTS program under RNTCP and so the contribution was made least (i.e., only 3%) for the diagnosis of TB from these centres.

Engagement of private practitioners in the functional referral system into the national program in India must be a priority to shorten delays and reduce the financial burden on families suffering from TB. A recent initiative in Chennai under the non-governmental organization Resource Group for Education and Advocacy for Community Health (REACH), along with the local health body and the National Institute for Research in Tuberculosis (NIRT), engaged more than 600 local private practitioners to participate in the RNCTP [32]. The scaling up of a public private mix (PPM) model has also occurred in 14 major cities of India [33] and resulted in early suspicion of illness and referral, reduced cost and increased participation in the RNTCP [4,34].

The REACH model identifies the potential private hospitals/clinics and provides a training program to the medical doctor and laboratory technician according to the RNTCP protocol. The model also offers a diagnostic and treatment facilities for the patients who are identified in these PPM centres. It also engages local community volunteer to provide support during anti-tuberculosis treatment. REACH also undertakes a monitoring and evaluation process along with the RNTCP staff to ensure the accountability of the system [32].

Our study has some limitations. The sample is restricted to those who registered at government primary health centres, which could limit the generalizability of the finding to other settings, both government and private. As this was a secondary analysis, we were limited to the data previously collected; factors such as pre-diagnostic travel and accompanying personal costs were not available. While the interviews of patients occurred shortly after TB diagnosis, recall bias may have resulted, especially for details related to costs incurred and exact dates of health visits.

## Conclusion

Despite more than a decade of DOTS implementation in the state of Tamil Nadu, we found a larger proportion of rural TB patients in the study site visited private sector practitioners prior to DOTS under RNTCP than visited public health facilities. Also, our data show that patients who first sought care in the private sector incurred higher pre-treatment OOP direct medical costs and longer health system delays than those who first sought care at public facilities. Our study findings are relevant in the context of India’s Strategic Plan 2017–2025 to integrate private provider engagement and thereby reduce catastrophic costs and time delays for TB patients.
